# A Practice Platform for Systematic Development of Microsurgical Instrument Technique

**DOI:** 10.7759/cureus.1253

**Published:** 2017-05-16

**Authors:** Darius Bagli, Rakan Odeh, Frank Penna, Ethan Grober, Nicolas Fernandez, Armando Lorenzo, Lisa Satterthwaite, Adam Dubrowski

**Affiliations:** 1 Urology, The Hospital for Sick Children, University of Toronto; 2 Urology, University of Toronto; 3 Surgical Skills Centre, University of Toronto; 4 Emergency Medicine, Pediatrics, Memorial University of Newfoundland

**Keywords:** simulation, microsurgery, training, simulation program

## Abstract

Many surgical disciplines, particularly those specializing in the pediatric age group, use microsurgical instruments with the assistance of either optical loupe or microscope magnification to perform high precision surgical procedures. We developed a course consisting of two parts: Part 1 consists of low fidelity, inanimate exercises or training and practice platform, and part 2 employed a rat model. In this report, we describe and provide templates for the first part of the course, namely the practice platform as an integral set of six low-fidelity exercises, each focusing on a specific aspect of instrument handling required to master the later use of the instruments during actual microsurgery. This platform is made to systematically and efficiently improve the microsurgical skills of junior as well as advanced surgical trainees.

## Introduction

Many surgical disciplines, particularly those specializing in the pediatric age group, use microsurgical instruments with the assistance of either optical loupe or microscope magnification to perform high precision surgical procedures [1]. Microsurgical techniques are some of the most challenging techniques to acquire in surgery, based on the precision of manual skills required [2]. Despite the widespread requirement for microsurgical loupe skills in such surgical subspecialties such as pediatric urology, pediatric plastic and reconstructive surgery, and the proliferation of training courses for more technology intensive areas such as minimally invasive and robotic surgery, no systematic platform to aid in the establishment of technical microsurgical loupe fundamentals exists. Indeed, the anachronistic apprenticeship models of traditional surgical teaching have never addressed this need. Ideal training strategies should focus on psychomotor techniques and instrument handling, as well as engender a culture of ongoing practice and skill development that provides a foundation for subsequent mastering of actual surgical procedures and intraoperative decision-making. This is a given principal in the acquisition of any higher order psychomotor skill. The rationale for such training programs is that trainees must learn to “walk” before they can go anywhere safely and efficiently navigate a route. This is analogous to a musician studying scales and arpeggios or an athlete mastering various pitches, throws, jumps, or dives. Both are fundamental prerequisites to later performing a musical work or executing strategy in a competition, respectively [3-5].

We developed a course consisting of two parts: Part 1 consists of low fidelity, inanimate exercises or training and practice platform, and part 2 employed a rat model. In this technical report, we describe and provide templates for the first part of the course. The described training platform is an integral set of six low-fidelity exercises, each focusing on a specific aspect of instrument handling required to master the later use of the instruments during actual microsurgery. All exercises were designed to use the same instruments; the Castroviejo needle driver, fine micro forces, and micro scissors (Lawton). The exercises are designed to enable learning of different skills related to microsurgical dexterity, and although we have arranged and report them in a specific order, this order is not prescriptive and other users may choose a different order. 

The exercises were originally grouped as a short two-hour skills course administered to surgical trainees in a large pediatric academic health science center or university-based simulation laboratory. Since 2012, we have provided this course annually to advanced surgical trainees in pediatric urologic surgery at The Hospital for Sick Children (Toronto, Ontario). Also, physicians with similar surgical needs as well as other technologists or researchers who are interested in acquiring or improving their basic microsurgical instrument handling technique in an animal research setting are also target learners. During the annual course at The Hospital for Sick Children, an attending surgeon from the Department of Urology (usually the course creator and the author of this technical report, DB) supervises the trainees’ performance after giving instructions on how to conduct each exercise.

However, recognizing international financial constraints and the high economic demands required for establishing, sustaining, and disseminating surgical training courses, the frequency of such courses has been reduced and running them on regular bases has become challenging. More recently, we determined that the exercise set is intrinsically able to function in a self-directed manner, where learners can practice the skills independently at home, forming the basis of a novel practice platform for learning microsurgical instrument dexterity. The low fidelity nature of the materials and models described below makes them easy to assemble for use at home, a simulation center, or even an in-situ environment as a microsurgical practice platform where most practice is indeed intended to occur. We have also created a series of six short instructional videos on how to construct and assemble the paper-based models and demonstrate important “dos” and “don’ts” for each exercise.

The technical report that follows describes each of the exercises, the materials that are required to build the models, and the assessment tools. Therefore, our intention is for this practice platform to be a fully accessible and modifiable adjunct to any new or existing microsurgical training program.

## Technical report

Dr. D. Bägli (DB) from The Hospital for Sick Children initially developed the microsurgical model designs. These models are easily constructed from PowerPoint/PDF printable patterns, simple scissors, a pencil, ruler, transparent adhesive tape, and utilize grains of rice, as well as the plastic insert from the 7-0 suture package. These tools are therefore inexpensive and easily attainable.

The part of the course that utilizes inanimate models described in this report has two formats: instructor based and self-directed. The tools, exercises, and assessment rubrics used in both formats of the course are identical; however, whenever applicable, we describe how these two formats differ. For example, the instructor-based format is conducted over a period of two hours. Each of the six exercises is practiced for 20 minutes, starting with a brief description of the exercise by the expert mentor. The two-hour duration was developed based on pilot work showing that most learners can complete at least one repetition of each exercise in that period. Learners who are not able finish at least one repetition are asked to complete a remediation exercise on their own time in the simulation laboratory, at home, or a combination of both formats. Each exercise is designed to concentrate on the development of a skill or movement related to the instruments: needle driver, forceps, and scissor handling.

In the self-directed, or home-based format, the elements contained in the first two hours of practice are conducted at home; however, there were no time limits set for the home-based format as these would be impossible to enforce. Interactive videos are used as instructional materials, as it has been shown that interactive video-based instructions for the acquisition of technical skills are as effective as expert-based instructions [6-7]. The videos are considered interactive as the learners can pause, scroll forward and backward through the videos in order to view any parts of the modeled performances that they may have difficulty with. Subsequently, the learners practice their skills, and when they perceive that they are ready to be assessed, they videotape their performance and send 20-30 second sample video clips to an assessor via email or into a secure shared Dropbox folder. The means of recording are not prescriptive, but suggestions are made to use laptops or webcams, or smartphones mounted on a tripod. The only stipulation is that the field of view captured by the recording includes the models, the instruments, and the hands of the leaners from the elbows down. Once assessed as competent, the learners are invited for the two-hour practice on the rat model at the simulation center. The assessments are performed by a surgeon with experience in both the microsurgical techniques and assessment metrics used. For all new assessors we provide a number of calibration assessments, where they are paired with a more seasoned assessor; they assess three to five videoed performances, and their scores are compared and any discrepancies are discussed.

This technical report is designed to describe the first part of the curriculum: the simulation exercises (Table 1). The six discrete exercises are described with brief instructions. The first two exercises concentrate on suturing accuracy using the Castroviejo needle driver in the dominant hand assisted by the fine micro forceps in the non-dominant hand. The third and fourth exercises concentrate on the use of micro scissors. The fifth and sixth exercises are designed to develop finger wrist dexterity with the micro-forceps alone in both the dominant and non-dominant hands.

**Table 1:** Summary of all simulation exercises within the curriculum.

	Name of exercise (Equipment required)	Institutions
Exercise 1	Angled face suturing. (Castro & forceps)	Running stitch, needle driver rotation, needle re-load/finger stabilization. Only use existing hole.
Exercise 2	Parallel face suturing (Castro and forceps)	Running stitch, needle driver rotation, needle re-load/finger stabilization. Only use existing hole.
Exercise 3	Scissor cuts (Lawton)	Don’t pass point scissor, don’t cut short, cut along thin lines.
Exercise 4	Scissor cuts w/removal (Lawton & forceps)	Don’t pass point scissor, don’t cut short, cut along thin lines, cut either side thick line and remove.
Exercise 5	Rice grain (forceps)	Place one grain per square X 10 beginning bottom right. Sequentially advance each rice grain by 1 empty square, as a train, until all advanced by 1. Do not touch borders. Continue until upper left 10 squares occupied. Reverse. Repeat with non-dominant hand.
Exercise 6	Pass rice grain through hole (dominant and non-dominant hand)	Pick up rice grain, rotate forceps only with fingers. DO NOT rotate wrist or elbow. Pass grain through hole. Complete 15 grains. Repeat with non-dominant hand.

There are two main model types: suturing models and scissor-forceps models. An instructional video demonstrating the six models with key instructions has also been developed and is accessible to all participants of the course (Video 1).

**Video 1:** An instructional video demonstrating the six models and how to use them.

Exercise 1: angled face suturing model

**Figure 1:**
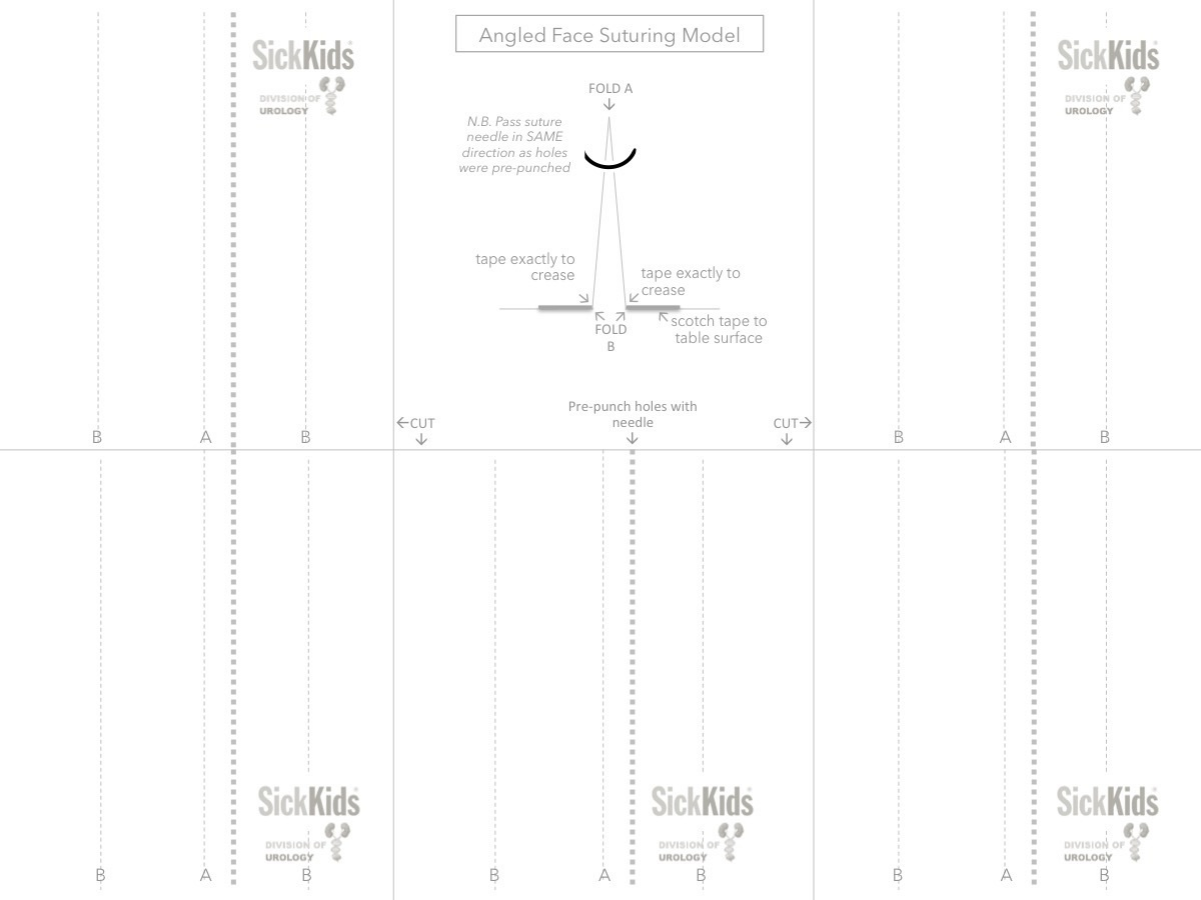
Model used for exercise 1. The middle panel depicts the model attached to the table where the curvature of the needle needs to be passed through the apex of the model through the printed and pre-punched holes. Each of the five models are made by cutting along the marked lines (CUT) and folded along the A and B lines. The base of the model is 1 cm wide (between folds marked with B).

The tools necessary to generate this model consist of ordinary printer paper, the Castroviejo needle driver and fine micro forceps. The paper should be folded along the indicated lines and taped to the table surface, and the target needle holes should be pre-punched. Figure 1 provides a template for creating these models. When printed out on standard letter format paper, the figure contains five models and a middle panel with instructions on how to cut, fold, and pre-punch the holes with a needle and adhere the models to a working surface.

Exercise

The trainee passes the needle through the prescribed target holes in the paper model emphasizing rotation of the needle driver. Specifically, it is emphasized that only the Castroviejo needle driver tip is to be manipulated rotationally in the direction of the arrow to accommodate the proper trajectory of the needle.

Assessment

**Figure 2:**
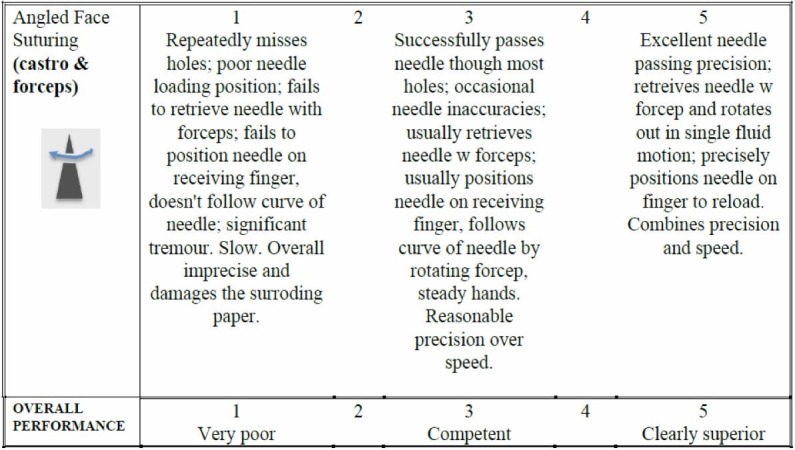
Assessment rubric for exercise 1.

Figure 2 provides a downloadable assessment rubric that is used for both the instructor and the learner for the home-based versions of the course. For the instructor-based version of the course, the rubric is used by the instructor/course lead during direct viewing of the performance. For the home-based versions of the course, the learner self-assesses the performance and is asked to submit a short clip via Dropbox for expert assessment.

Exercise 2: parallel face suturing model 

**Figure 3:**
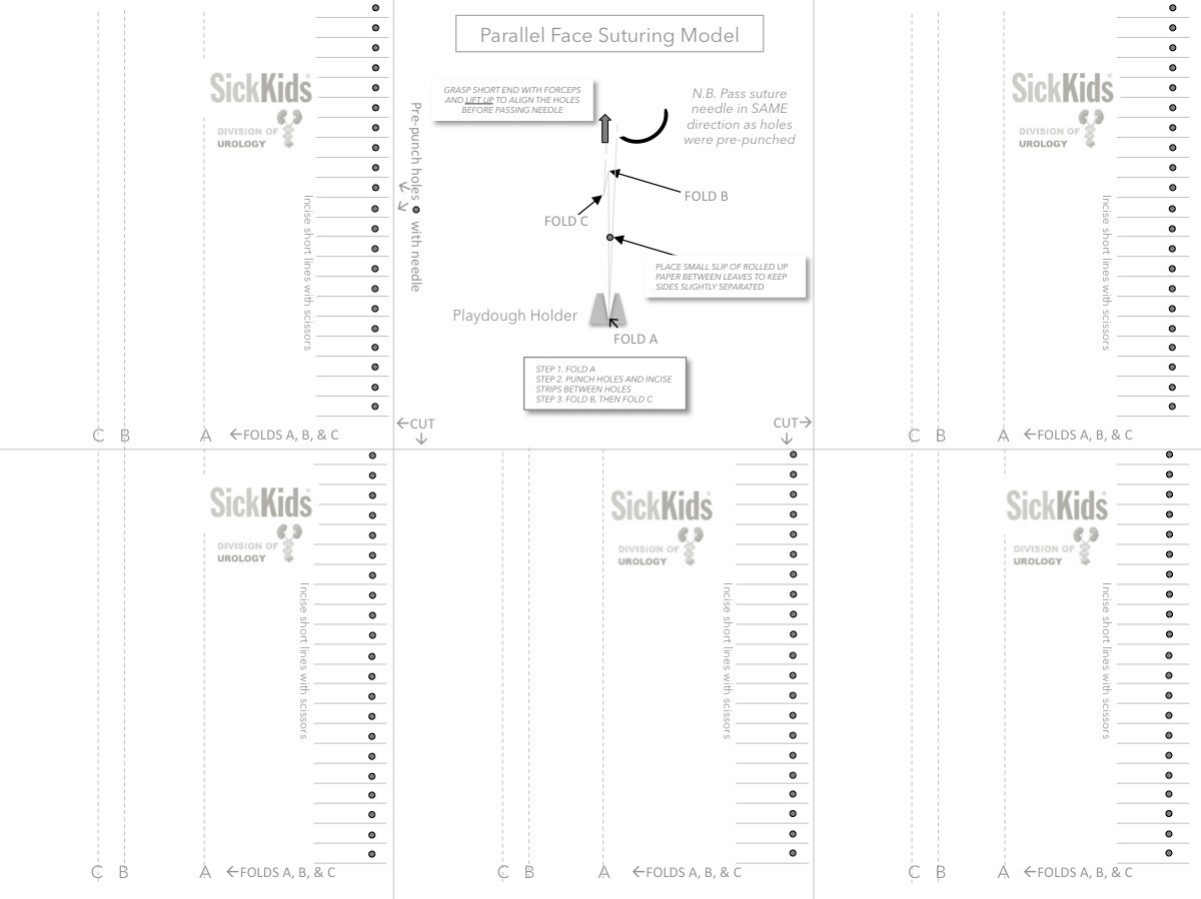
Model used for exercise 2. The middle panel depicts the model attached to the table where the curvature of the needle needs to be passed through the leafs at the edges of the model through the printed and pre-punched holes. Each of the five models are made by cutting along the marked lines (CUT) and folded along the A, B, and C lines. Note that the folds B and C create an offset to shift the two suturing edges to create an extra dimension. The base of the model is attached to the surface of the table with Play-Doh.

The tools necessary to generate this model and conduct the exercise consist of two ordinary sheets of printer paper, first with holes pre-punched, and with holes now aligned, cut into individual facing pairs of tags (cutting between the holes) (Figure 3). Each tag can be grasped with aid of forceps to facilitate needle passage through both holes, thus simulating stabilizing or positioning small portions of actual tissue to accommodate the needle targeting.

Exercise

The trainees need to pass the needle through the prescribed target holes in the paper model allowing for forceps to be used to move the “tissue” to coordinate needle placement.

Assessments

**Figure 4:**
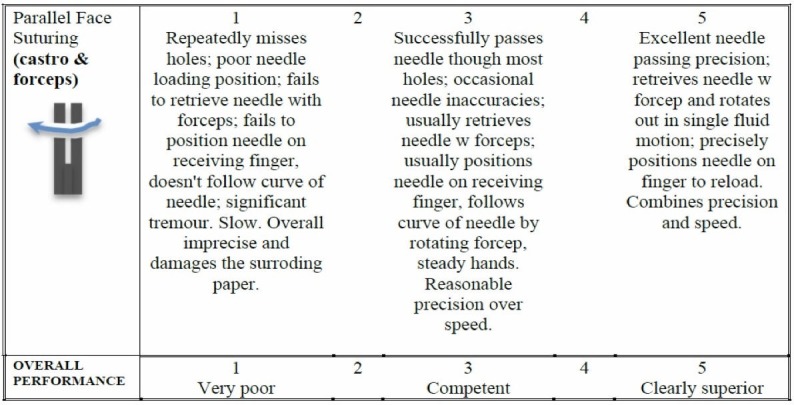
Assessment rubric for exercise 2.

Figure 4 is a downloadable assessment rubric that is used for both instructor and home-based versions of the course. Just as in Exercise 1, for the instructor-based version of the course, the rubric is used by the instructor/course lead during direct viewing of the performance. For the home-based versions of the course, the learner self-assesses the performance and is asked to submit a short clip via Dropbox for expert assessment.

Exercise 3: scissor cuts

**Figure 5:**
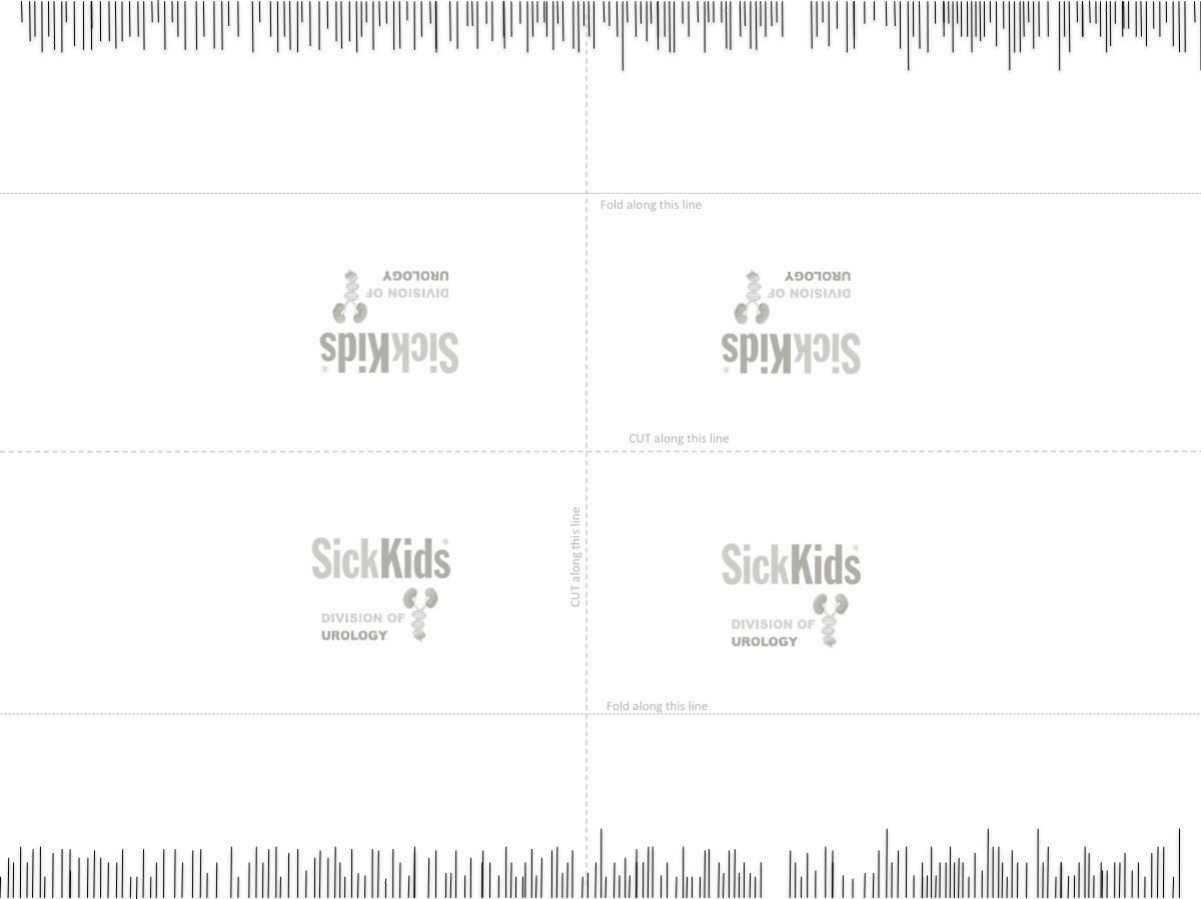
Model used for exercise 3. This downloadable figure creates four models. Cut and fold along the marked lines. Attach the model with a tape to the working surface. Cut the lines marked on the paper as accurately and as fast as possible.

The tools necessary to generate this model and conduct the exercise consist of a sheet of printer paper pre-lined as shown in Figure 5, Lawton tenotomy scissors, and micro forceps.

Exercise

The trainees are required to cut exactly on different length lines without cutting off from the lines, undercutting (stopping short), or pass-pointing (cutting past the line).

Assessment

**Figure 6:**
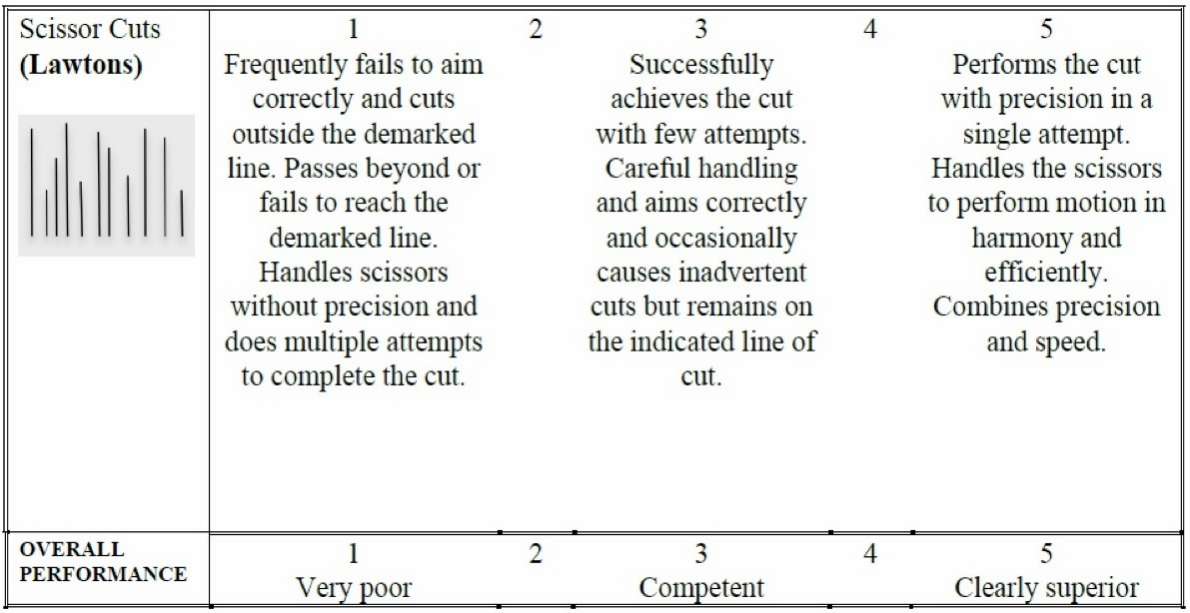
Assessment rubric for exercise 3.

Figure 6 is the assessment rubric that is used for both the instructor and the self-directed versions of the course. Just as in the other exercises, for the instructor-based version of the course, the rubric is used by the instructor/course lead during direct viewing of the performance. For the home-based versions of the course, the learner self-assesses the performance and is asked to submit a short clip via Dropbox for expert assessment.

Exercise 4: scissor cuts with “tissue” removal

**Figure 7:**
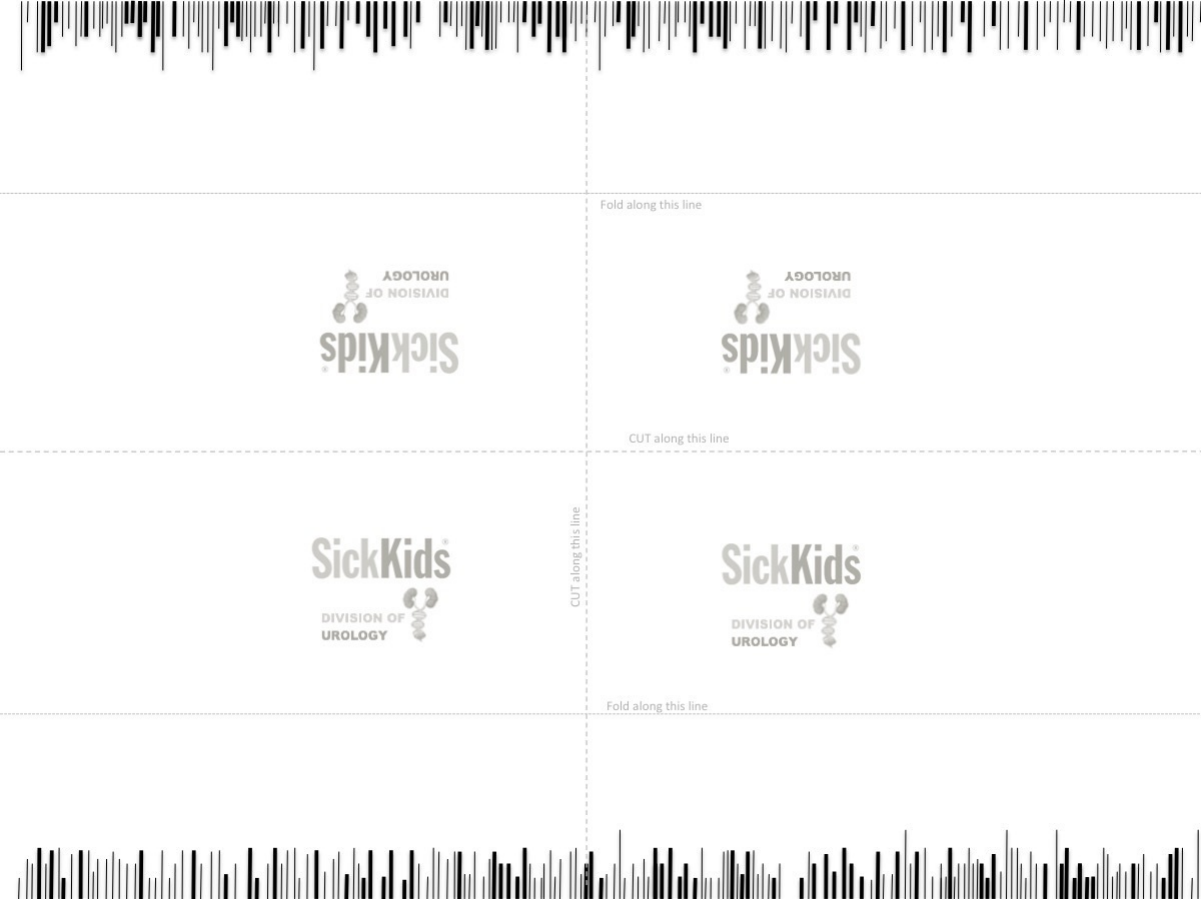
Model used for exercise 4. This downloadable figure creates four models. Cut and fold along the marked lines. Attach the model with a tape to the working surface. Cut the lines marked on the paper as accurately and as fast as possible. Remove the colored portions between the cuts.

The tools necessary to generate this model and conduct the exercise consist of a sheet of printer paper lined with thick and thin lines as shown in Figure 7, Lawton tenotomy scissors, and micro forceps.

Exercise

The trainees are required to cut exactly on different length fine lines without cutting off from the lines, undercutting or pass-pointing, as well as cut on either side of the thick lines with subsequent removal of the thick-line strip.

Assessment

**Figure 8:**
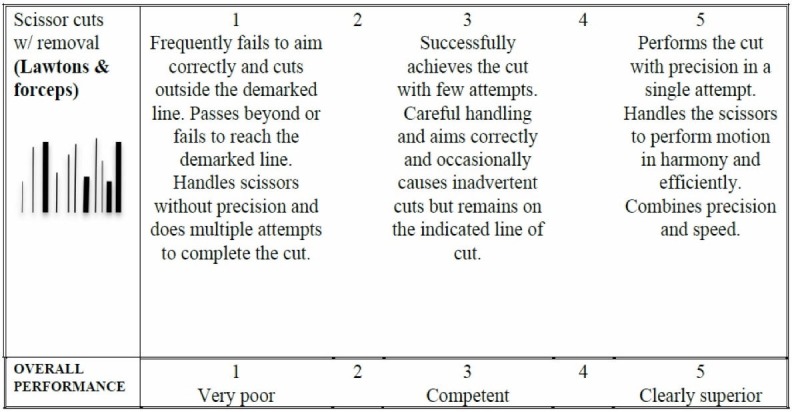
Assessment rubric for exercise 4.

Figure 8 is the assessment rubric that is used for both the instructor and self-directed versions of the course. Just as in the other exercises, for the instructor-based version of the course, the rubric is used by the instructor/course lead during direct viewing of the performance. For the home-based versions of the course, the learner self-assesses the performance and is asked to submit a short clip via Dropbox for expert assessment.

Exercise 5: rice grain transfer with forceps

**Figure 9:**
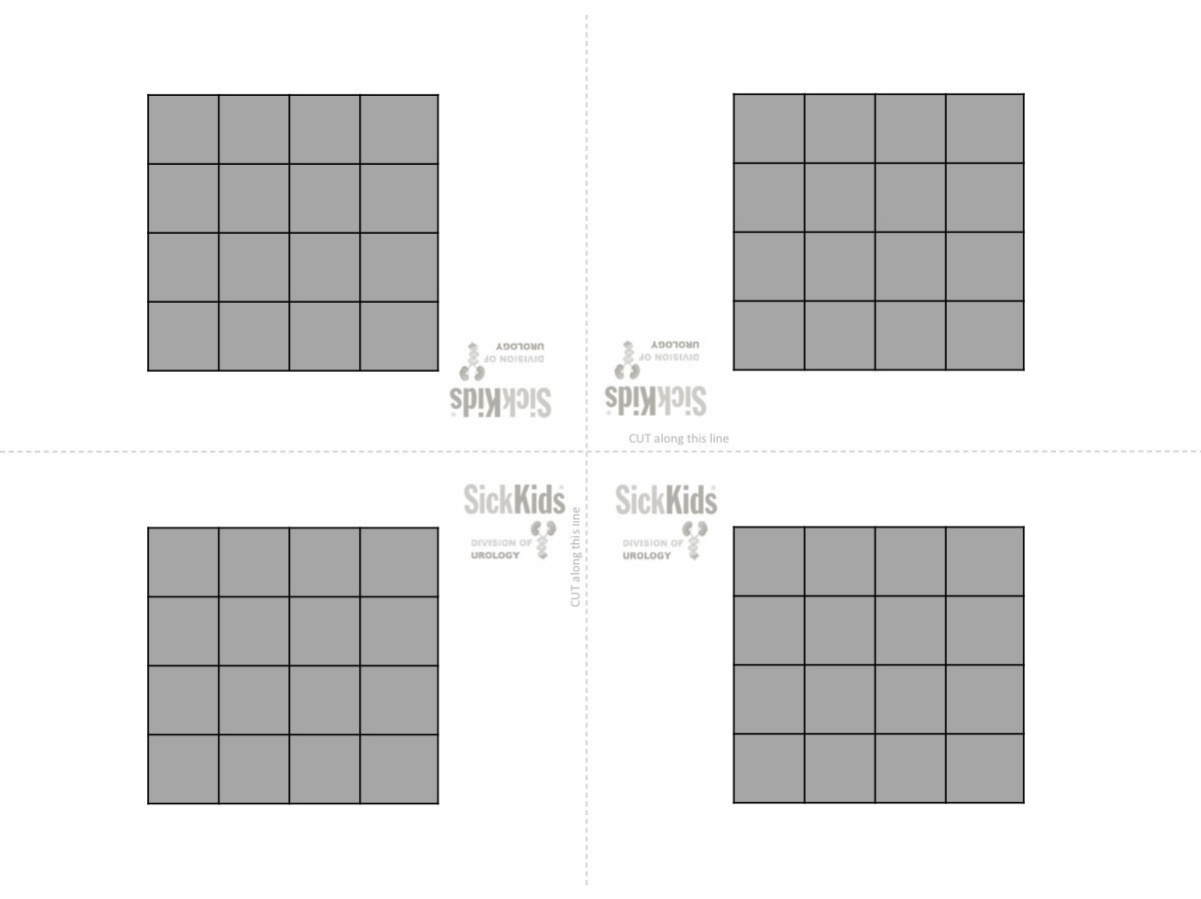
Model used for exercise 5. This downloadable figure creates four models. Cut along the dashed lines.

The tools necessary to generate this model and conduct the exercise consist of a sheet of printer paper pre-printed with a 4 x 4 square grid (Figure 9) and micro forceps.

Exercise

The trainees are required to place 10 grains of rice on to the first ten squares, transfer the grains of rice, one at a time, in sequence around the 4 x 4 matrix of squares. The nature of a grain of rice requires the use of precise pressure to grasp it with the forceps; too little pressure and the grain will be dropped, excessive pressure and the grain will be ejected from the forceps. This should be performed with the dominant, followed by the non-dominant hand.

Assessment

**Figure 10:**
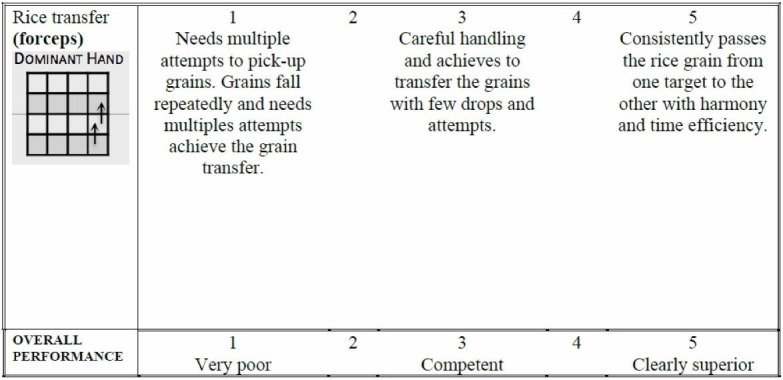
Assessment rubric for exercise 5.

Figure 10 is the assessment rubric that is used for both the instructor and the self-directed versions of the course. Just as in the other exercises, for the instructor-based version of the course, the rubric is used by the instructor/course lead during direct viewing of the performance. For the home-based versions of the course, the learner self-assesses the performance and is asked to submit a short clip via Dropbox for expert assessment. Note that for this exercise both dominant and non-dominant hand performances are scored with separate but identical assessment rubrics.

Exercise 6: pass rice grain through hole with forceps

**Figure 11:**
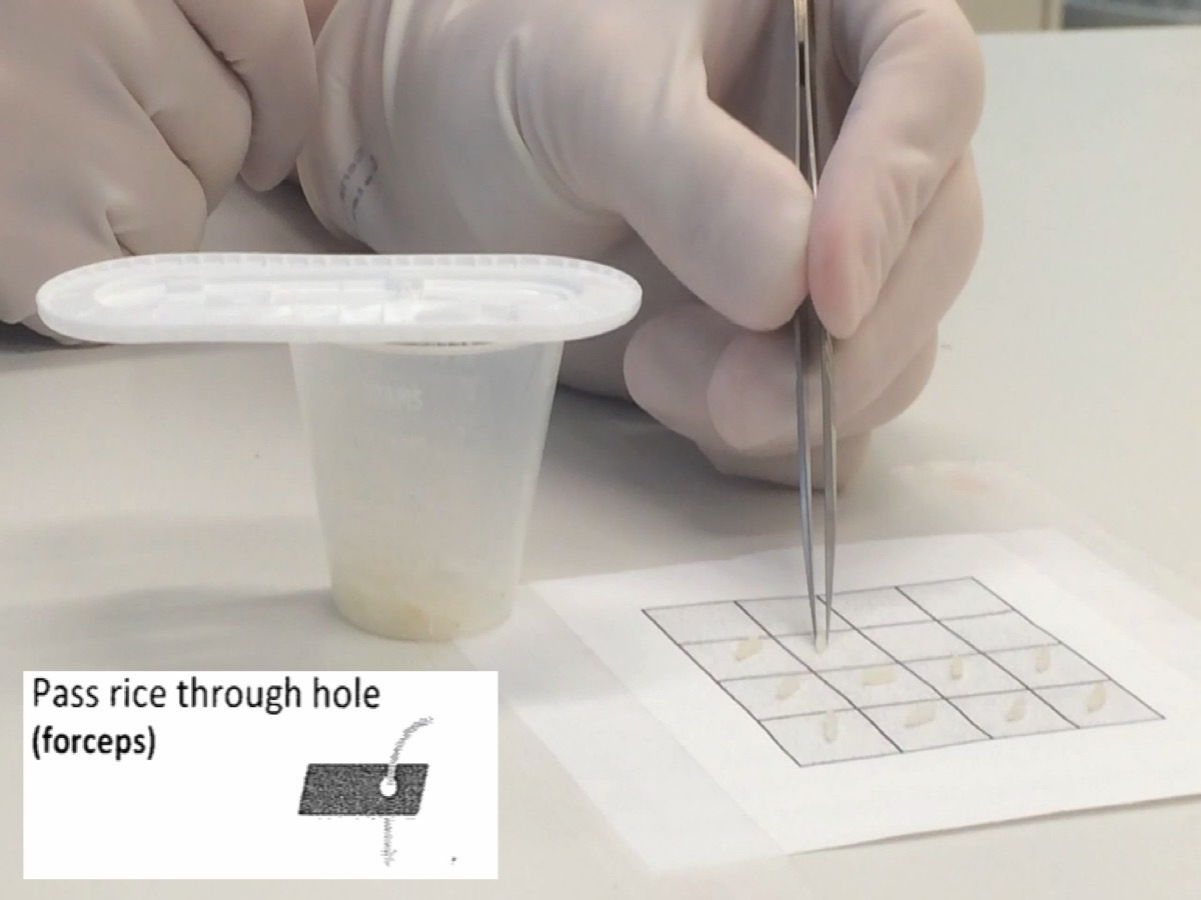
A photograph of the medicine cup and insert set up for exercise 6.

The tools necessary to generate this model and conduct the exercise consist of a small medicine cup, the plastic insert from a 7-0 suture package, rice, and micro-forceps (Figure 11).

Exercise

The trainee is asked to place the plastic insert onto the medicine cup with the largest hole extending off from the cup. Specifically, the trainee picks up one rice grain at a time, rotates the forceps and the rice grain 180 degrees with the fingers only on the long axis of the instrument, without dropping or jettisoning the rice grain and places it through the hole.

Assessment

**Figure 12:**
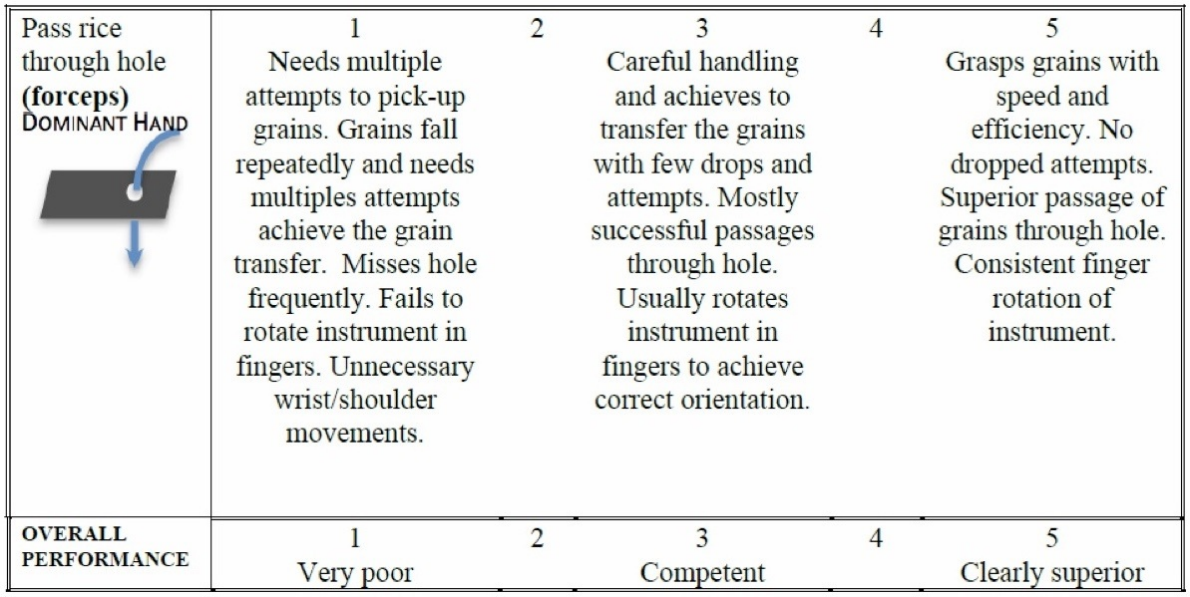
Assessment rubric for exercise 6.

Figure 12 is the assessment rubric that is used for both the instructor and the self-directed versions of the course. Just as in the other exercises, for the instructor-based version of the course, the rubric is used by the instructor/course lead during direct viewing of the performance. For the home-based versions of the course, the learner self-assesses the performance and is asked to submit a short clip via Dropbox for expert assessment. Note that for this exercise both dominant and non-dominant hand performances are scored with separate but identical assessment rubrics.

## Discussion

The goal of this technical report is to describe and provide easily accessible materials and templates constituting a practice platform designed to develop the fundamental skills related to microsurgical instrument handling performance. It is part of a surgical course divided into two parts: simple, bench top training platform and animal (rat) model simulation. In this technical report, we describe in detail the practice platform utilizing the bench top training platform. The overall goal of the six exercises described is to create a systematic practice platform to achieve and master technical competence in the handling of basic microsurgical instruments among surgical trainees. The low fidelity nature of the exercises allows for easy and highly cost effective construction of the required models. The motivation is to encourage and discipline dozens to hundreds of accumulated hours of practice to master the basic target skill for each instrument.

In addition, this part of the course can be delivered in instructor-based, or self-directed formats. In the instructor-based format, the instructor demonstrates the skills, provides feedback, and assesses the proficiency using the provided assessment rubrics. In the self-directed formats, interactive, instructional videos are used as a form of instruction. The learners practice independently at home or in another suitable setting free from distractions. When trainees have self-assessed that they have attained a target level of proficiency [8], they video record their performance and send sample video clips to the expert via email or into a secure shared cloud-shared folder. The expert assesses their performance using the same assessment rubrics as in the instructor-based format. Once the trainees are assessed as proficient (i.e. >3/5 on all assessment rubrics), they are invited to take part in the second part of the course where they implement their skills on a rat model.

Although a detailed description of the rat model is outside of the scope of this technical report, we provide herein a brief description. Here, an “open urethral plate” (simulating the analog of the common human infant genital maldevelopment of hypospadias) is developed from the rectus muscle of the anesthetized animal, using two parallel incisions approximately 4 cm long and 9-10 mm apart. The open urethral plate is a universally encountered feature of common genital reconstruction in children. The edges of these two incisions are then sewn together over a small silicone tube used as a guide, thereby recreating a simulated human infant “urethra” from living tissue.

Future studies will be required to determine the impact of mastering basic instrument techniques on subsequent performance of actual procedures on rats or human patients and ultimately on clinical surgical outcomes. However, evidence in non-medical disciplines demonstrate that deliberate and accumulated practice enhances execution of skills and the learners’ confidence. The exercise set is currently being developed into a freestanding self-administered web-based course of practice, wherein participants can upload their video samples of their progress for rating by surgical experts. The use of crowd-sourced evaluation is also being considered.

## Conclusions

This platform is made to systematically and efficiently improve the microsurgical skills of junior as well as advanced surgical trainees. The low fidelity nature of the practice platform allows easy construction and performance in a repetitive fashion, allowing for the all-important accumulation of practice hours. From an economics point of view, this platform is well positioned to minimize the teaching budgets needed for the regular trainees practice. The scoring rubrics described will allow trainees to rate themselves and document the nature and pace of their improvement and learning. An ongoing next step is the translation of this platform for web-based access, wherein trainees will upload their video clips of their performance to be rated by our staff members at The Hospital for Sick Children.
